# Amphiphilic Janus Particles Confined in Symmetrical and Janus-Like Slits

**DOI:** 10.1021/acsomega.3c01180

**Published:** 2023-05-16

**Authors:** Łukasz Baran, Małgorzata Borówko, Wojciech Rżysko, Jakub Smołka

**Affiliations:** † Department of Theoretical Chemistry, Institute of Chemical Sciences, Faculty of Chemistry, Maria Curie-Sklodowska University in Lublin, Pl. M Curie-Sklodowskiej 3, 20-031 Lublin, Poland; ‡ Department of Computer Science, Lublin University of Technology, Nadbystrzycka 36B, 20-618 Lublin, Poland

## Abstract

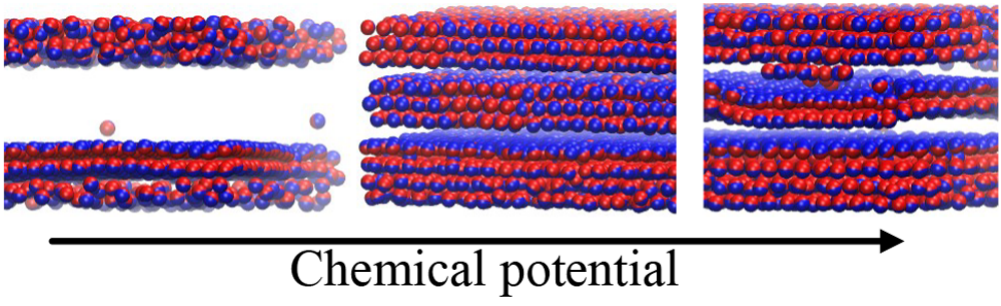

We use Monte Carlo simulations to investigate the behavior of Janus spheres composed of attractive and repulsive parts confined between two parallel solid surfaces. The slits with identical and competing walls are studied. The adsorption isotherms of Janus particles are determined, and the impact of the density in the pore on the morphology is discussed in detail. So far, this issue has not been systematically investigated. New, unique structures are observed along the isotherms, including the bilayer and three-layer structures located at different distances from the walls. We analyze how selected parameters affect the positional and orientational ordering in these layers. In some cases, the particles form highly ordered hexagonal lattices.

## 1 Introduction

Self-assembly of nanoparticles is a topic attracting intense scientific interest. Various strategies have been developed for the design of novel structures with desired properties, geometries, and dimensions.^1−4^ These modern materials have a wide range of applications in electronics, photonics, drug delivery, medical diagnostics, and sensors to name but a few. In this respect, Janus particles (JPs) with two different domains on their surfaces represent an important field of research. Numerous experimental studies of JPs have been complemented by a range of theoretical works devoted to the analysis of self-assembled structures and phase diagrams.^5−15^

Interactions between the JPs depend on their spatial separation and orientation, resulting in a great variety of self-assembly scenarios. Considerable research effort has been devoted to studying the self-assembly of spherical Janus particles that interact via different potentials.^5−10,16^

The interactions can be modeled using an effective anisotropic potential involving the orientation vectors defined by the symmetry axis of the spheres.^6^ In other models, JPs are treated as a kind of patchy particles decorated with domains on the surfaces exerting attractive forces. The most popular is the Kern-Frenkel model with one attractive patch on an inert (repulsive) surface.^7^

Self-assembly of JPs in a one-component bulk system depends on the assumed interaction model, density, and temperature. Depending on the conditions JPs can assemble into aggregates of various sizes and shapes, vesicles, bilayers, ordered 2D arrays, and 3D crystals.^1−3,5−9,11−14^ It has been proved that the Kern-Frenkel model generates the formation of micelles, vesicles, bilayers, and different crystal phases.^12−14^ Granick’s group^2,3,5^ has shown that colloidal spheres, hydrophobic on one hemisphere and charged on the other, assemble into small compact aggregates or wormlike strings. Rosenthal et al.^6^ have studied the aggregation of amphiphilic Janus particles in bulk phases and shown that an increase in the system density causes the formation of different aggregates, from spherical micelles, through icosahedrons to huge elongated structures. The recent simulations^15^ have suggested a simple route toward 2D crystals via the direct self-assembly of 3D systems of amphiphilic JPs that spontaneously formed ordered bilayered lamellae. Within each layer of the bilayered lamellae, abundant highly ordered 2D crystals including the Frank–Kasper phases and open kagome lattice were observed.^15^

The behavior of JPs can be also manipulated by external magnetic or electric fields,^1^ and interfaces^17^ the presence of different solvents or isotropic nanoparticles.^18−21^ Another method for the design of unique structures is also to assemble JPs under confinement. This can be realized by the adsorption of JPs on solid surfaces or in different pores.^8,9,22−36^ Recent advances in computer simulations of colloidal self-assembly driven by anisotropic or orientation-dependent interparticle interactions are highlighted in the comprehensive review.^16^

A two-dimensional system involving JPs can be considered as a monolayer formed on an inert substrate. Monte Carlo simulations of Janus disks have shown how the assembly and phase transitions depend on the strength of interaction between different patches.^8,9^ The self-consistent phonon theory has been applied to study two-dimensional crystalline phases of amphiphilic JPs.^22^ This approach predicts the formation of zigzag stripes, trimers, and rotationally disordered plastic crystal phases. The orientational ordering of two-dimensional closely packed JPs has been also explored by extensive Monte Carlo simulations.^23^ For large enough patch sizes, the system exhibited a second-order transition into a phase with the stripe patterns of the patches breaking the threefold rotational symmetry. The transition belongs to the universality class of the three-state Potts model. Quite recently, Sato^24^ has applied MC simulations to study how two-dimensional self-assemblies formed by the one-patch Kern-Frenkel particles on a flat plane depend on the interaction length and patch area. Huang et al.^37^ studied the melting and solid–solid transitions of two-dimensional crystals composed of Janus colloidal spheres interacting via anisotropic attractive pair potential proposed by Doye et al.^10^ They discovered a first-order solid–solid transition from a single crystal with uniform stripes to a novel crystal with polycrystalline domains of stripes. In this transition, the particles lost their long-range orientation order but maintained their crystalline positional order. Equilibrium self-assembly of inverse patchy colloids in two dimensions is investigated using Monte Carlo method.^38^ Various ordered structures were obtained by tuning the size of the single patch, density, and interaction strengths.

Numerous studies have shown that the structure of the adsorbed layer can be controlled by the surface potential.^25−34,36,39^ The behavior of amphiphilic JPs at planar walls has been investigated using classical density functional theory.^25^ In this case, inert surfaces and walls preferring the hydrophilic patch cause the formation of bilayers, while hydrophobic walls typically induce competition between mono- and bilayer structures. Multilayer adsorption of JPs has been also discussed in the framework of the lattice model.^26^

From a cognitive point of view, it is very interesting to discuss the self-assembly of JPs in different pores.^28−35,39^ Several theoretical works dealt with the behavior of JPs in two-dimensional channels.^28−30^ Molecular dynamic simulations were carried out for Janus disks in a two-dimensional plane, which were trapped in a one-dimensional harmonic potential,^28^ and for Janus spheres in a circular symmetric harmonic trap.^29^ In these systems, various membrane-like morphologies were found. These structures consist of single and multiple chain configurations with different orientations of the particles with respect to each other,^28^ multiple helices, and Bernal spirals.^29^ The assembly of amphiphilic JPs in two-dimensional slits has been investigated using Monte Carlo simulations.^30^ The slits with identical walls and Janus-like pores were investigated. The study has shown how interactions between particles, the nature of “walls”, and their separation influence self-assembly.

The behavior of JPs in three-dimensional channels has been studied using density functional theory^31,32^ and molecular simulations.^33−36,39^ Rosenthal and Klapp^31^ have investigated amphiphilic JPs in slits and discussed the competition between planar structures preferred by the surfaces and nonplanar (micellar) structures induced by particle–particle interactions. They proved that the surfaces can stabilize bilayer structures characterized by a high degree of ordering relative to the walls. Iwashita and Kimura^33,34^ have explored the behavior of JPs under confinement. In the first stage, they analyzed purely orientational ordering in close-packed one-patch particles confined in narrow slits and confirmed the decisive impact of confinement on the ordering of JPs.^33^ Then, they discussed the impact of the density on the orientational ordering in the slits.^34^ Kobayashi and Arai^35^ have employed dissipative particle dynamics simulation to investigate the phase behavior of JPs with hydrophobic hemispheres in nanotubes with different walls—hydrophobic, hydrophilic, and hydroneutral. They have found the assemblies that do not occur in bulk systems. Various unique structures have been also observed in Monte Carlo simulations of JPs confined in different slits.^36^ Several interesting phenomena were observed in such systems, namely, two-dimensional “crystallization of clusters”, surface-induced formation of “levitating” slabs, and “inverted” adsorption in Janus-like pores.^36^ Clusters formed by dumbbell-like JPs confined in thin space between two parallel walls have been also discussed.^39^

To sum up, numerous studies revealed that JPs confined in nanospaces form unique structures that are not observed in their bulk counterpart. The self-assembly of JPs in pores results from a competition between particle–particle interactions and particle–wall confining forces. This process depends on many parameters, such as the geometry of pores, nature of walls, type of JPs, density, and temperature. Despite intensive research, many questions regarding the role of individual parameters remain unanswered. For example, the evolution of the structure with increasing pore filling has not been systematically discussed. Furthermore, the mechanism of crystallization of JPs under confinement has not yet been clarified. The interplay between the aggregation of JPs and their adsorption at the pore walls also requires further studies.

Herein, we aimed to gain fundamental insights into the intricate relationship between the slit characteristics, the interactions between JPs, the system density, and the resulting system morphology. We study amphiphilic JPs in different slits using Monte Carlo simulation in a grand canonical ensemble. We investigated two types of slits—symmetrical ones and Janus-like pores.

The organization of the paper is as follows. In Section 2 we introduce the model, briefly describe our simulation method, and define the calculated quantities. Then, in Section 3 we report and discuss our results. Finally, in Section 4 we conclude the paper with a few remarks.

## 2 Methods

### Model

We study the behavior of Janus particles confined in different slits. The wall separation is assumed to be *L*_*z*_. The model of amphiphilic Janus particle used here is essentially the same as described in earlier works.^25,35,36,39^ The Janus particles consist of two hemispheres; the parts named *A* are attractive while the parts *R* are repulsive. The interaction potential between two Janus particles is expressed as a sum of the hard-sphere potential, *v*_*JJ*_^*hs*^, and the anisotropic contribution
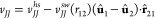
1where the vectors **r**_1_ and **r**_2_ characterize the positions of the interacting particles, *r*_12_ = |**r**_12_| = |**r**_1_ – **r**_2_|, , and ,  are the unit patch orientation vectors; the vector  points toward the repulsive (*R*) part of the particle *i* (*i* = 1, 2), and the  is the square-well potential, with the depth ε_*JJ*_ and the range 1.6σ. The Janus particles prefer to be oriented in opposite directions, such that the attractive parts, *A*, point toward one another, while the antiparallel orientation with facing *R*-sides is the most energetically unfavorable one. The parallel configurations are energetically neutral.

The energy of interactions of JPs with both walls is the following sum
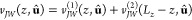
2while *v*_*JW*_^(*i*)^ is the potential of interaction with the *i*th wall
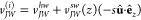
3where *v*_*JW*_^*hw*^ is the hard-wall potential,  is the unit vector along the axis *z*, perpendicular to the surface, and *v*_*JW*_^*sw*^ is the square-well potential of the well range between 0.5σ and 2.5σ, and of the depth ε_*JW*_. The factor *s* = ±1 characterizes the wall. When the wall attracts the *R*-parts of JPs (and repels their *A* sides), we set *s* = 1 and abbreviate such a surface as WR. For *s* = −1, however, the *A*-sides of JPs are attracted by the wall (WA).

We study two types of slit-like pores: (i) symmetrical slits (SP) with the same walls (WR) and (ii) Janus-like (antisymmetrical) slits (AP), in which one wall is of the WR-type, while the other is the WA-wall.

The Janus particle diameter σ_*J*_ = σ is the unit of distance, and ε_*JJ*_ = ε is taken as the unit of energy. In this work, we use the reduced parameters, e.g., z* = *z*/σ, *L*_z_^*^ = *L*_*z*_/σ, or ε_JW_^*^ = ε_*JW*_/ε, etc.

Our model was built based on real systems. The potential (eq 1) mimics interactions between JPs composed of hydrophobic (A) and hydrophilic (R) parts in aqueous solutions. The water molecules are adsorbed at the hydrophilic patches of the particles. Due to the resulting steric exclusion, the hydrophilic portions of JPs repel each other. At the same time, configurations with hydrophobic lobes facing each other are preferred because they minimize contact with water in this way. This means that the hydrophobic sides are effectively attractive. A classical example is silica particles, one hemisphere of which is covered with gold, immersed in an aqueous solution.^40,41^ On the other hand, the WR face may represent polar substrates such as bare silicon, while the wall of WA mimics nonpolar surfaces such as a methylene-capped silicon surface.^42^ However, a great variety of one-patch particles can be represented by the model considered, for example, gold nanoparticles coated with single-stranded DNA^43^ and others.^44^

### Simulation Details

We have carried out a series of Monte Carlo simulations in the grand canonical ensemble.^45,46^ Each Monte Carlo step consisted of the following attempts to change the state of the state of the system: a mutual change of the position and orientation of a randomly chosen particle, its reorientation, a “jump” (the annihilation of a selected particle, followed by the creation of a particle at a new position and a new orientation), and an attempt to insert or remove a particle from the system. The use of various ways to change the state of the system increases the efficiency of the method. An acceptance of removal or insertion leads to a change in the number of particles in the system. For other attempts to change the state of the system, the density remains constant. Annihilations, creations, and jumps allow us to avoid ”arresting” the system in metastable states and facilitate the exploration of the free energy landscape. Moreover, in dense systems, certain ”moves” make it easier to fit into the equilibrium structure (e.g., reorientation of a particle without changing its position). The jumps can improve the removal of lattice defects in crystals. Long simulation runs (on the order of 10^9^ MC steps) were necessary to reach equilibrium. The production runs lasted not less than 10^7^ MC steps. To estimate the error bars, we performed five independent simulations.

### Calculated Observables

During simulations, we have collected density profiles, orientations of JPs, and energies of interactions between the particles and with the walls and used them to calculate various characteristics of the systems.

To measure the adsorption magnitude, we determine the effective density of JPs in a slit
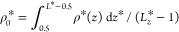
4

In order to analyze the orientation of JPs with respect to the walls, we used the order parameter characterizing the local “polarization”, *h*(*z*), defined as^25^
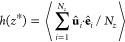
5where *N*_*z*_ is the number of particles within a slice of width Δ*z*. The order parameter assumes values between −1 and 1. For *h* = 1 (*h* = −1), the R-parts of all JPs point toward the lower (upper) wall.

To characterize local structures that evolved during the simulation we use the parameters based on the concept of the local-bond-orientational parameters proposed by Steinhardt et al.^47^ According to this method, a quantitative measure of structure around a particle *i* is characterized by the bond (a vector connecting neighboring particles *i* and *j*) order parameter
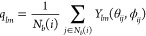
6where *N*_*b*_(*i*) is the number of neighbors of particle *i*, θ_*ij*_ and ϕ_*ij*_ specify the orientation of the bond between particles *i* and *j*, and *Y*_*l*_^*m*^ are the spherical harmonics. We assume that the Janus particles are considered to be coordinating if their distance is less than 1.6σ.

Recently, Muller-Plathe’s group^48,49^ has proposed new order parameters that make possible the identification of quasi-two-dimensional Kagome and hexagonal phases
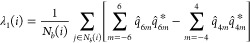
7and
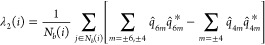
8where
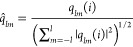
9and an asterisk denotes the complex conjugation. In the order parameter space (λ_1_, λ_2_), a given phase is represented by the point (λ_10_, λ_20_). The parameters which identify the Kagome and hexagonal phases correspond to the points (0.65, 0.7) and (0.0, 0.8), respectively. We assume that a particle belongs to a given phase if the λ_1_ and λ_2_ have the values λ_*i*_ = λ_*i*0_ ± δ_*i*_, where δ_*i*_ = 0.1λ_*i*0_ and *i* = 1, 2.

We define the parameter characterizing a degree of the ordering in a given layer as
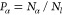
10where *N*_α_ is the number of particles that satisfy the assumed conditions (α = Kagome, hex) and *N*_*l*_ is the number of particles in a layer.

In the following, we will refer to the wall separation as H* = *L*_z_^*^.

## 3 Results and Discussion

### Description of the Considered Systems

We report the results of simulations for JPs adsorbed in the relatively narrow slits with the wall-separation H* = 7 and the wider pores with H* = 11. For these values of H*, the force fields of walls do not overlap inside the slits. Nevertheless, we show that for the assumed range of interparticle interactions, both walls affect the internal structure of the adsorbed fluid. The pore is in equilibrium with the bulk reservoir in which the chemical potential of JPs is equal to μ*. Simulations were performed at temperature T* = 0.2. We investigate symmetrical (SP) and Janus-like (AP) pores. Moreover, two cases of particle–wall couplings are considered: weak (WC) and strong (SC) interactions, with ε_JW_^*^ = 2 and ε_JW_^*^ = 4, respectively.

We assumed the parameters similar to that used in earlier works.^30,31,36^ In particular, we previously considered the Janus particles in narrow pores with overlapping force fields generated by the walls.^36^ The behavior of these particles in wider pores has not been the subject of research until now.

Our goal is to show how an increase in density changes the morphology of these systems. We also analyze the influence of a slit type and the strength of particle–wall interactions on the system structure. We are interested in the mutual interplay between anisotropic particle–particle and particle–wall interactions. The effect of such competition is considerable when the strength of these interactions is similar. Therefore, we focus on the case of weak walls (WC). To limit the size of the article, we describe only the most representative and significant results obtained with stronger walls (SC). For very strong walls the system structure could be completely dominated by the surfaces.

In some of the studied systems, a hexagonal phase was detected, and the values of the parameter *P*_*hex*_ are collected in tables presented in Supporting Information (SI).

### Adsorption and Self-Assembly in the Narrow Slits with H* = 7

**Figure 1:**
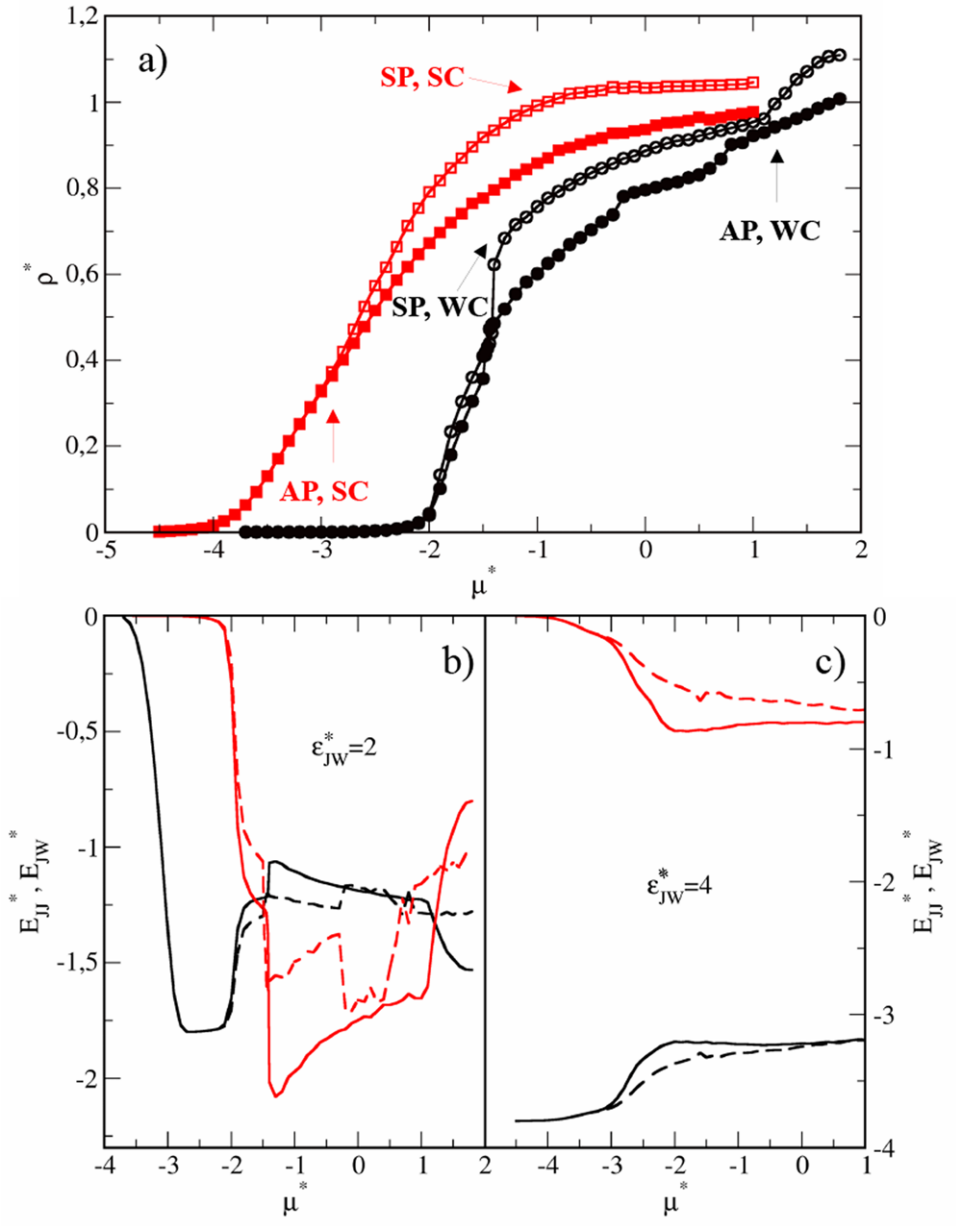
Results for the narrow pores (H* = 7). (a) Adsorption isotherms for the case WC, ε_JW_^*^ = 2 (black circles) and SC, ε_JW_^*^ = 4 (red squares) in symmetrical pores SP (open symbols) and Janus-like pores AP (filled symbols). The energies *E*_JJ_^*^ (red lines) and *E*_JW_^*^ (black lines) in symmetrical pores (solid lines) and Janus-like pores (dashed lines) for the case WC, ε_JW_^*^ = 2 (b) and SC, ε_JW_^*^ = 4 (c).

We begin with the analysis of the results for the case of weak JP–wall interactions (WC). The adsorption isotherms obtained for the studied narrow slits are presented in Figure 1. Adsorption is stronger in symmetrical pores than in Janus-like pores. In the range −2.2 < μ* < −1.4 the densities in SP and AP are similar and quickly, but gradually, increase. However, at μ* ≈ – 1.4 the densities rapidly jump. The jump is much greater for the symmetrical pore. With a further increase in the chemical potential, the density of adsorbed JPs continuously rises. For the symmetrical pore, at μ* ≈ 1.0 the increase accelerates. In the case of a Janus-like pore, we see two inflection points–at μ* ≈ – 0.2 and at μ* ≈ 0.8.

It is instructive to analyze the energies of the particle–wall  and particle–particle  interactions along the isotherms shown in Figure 1b. The energies of particle–wall interactions gradually elevate as the chemical potential increases to μ* ≈ −1.4 where the small steps are visible. At the same point, a rapid decrease in particle–particle interaction energies is observed. These jumps are more profound for the SP than for the AP. Then, the functions  and  are different for symmetrical and antisymmetrical slits. For SP, the *E*_JW_^*^ slightly decreases while the *E*_JW_^*^ considerably increases. However, at μ* ≈ 1.2 the trend is reversing; the *E*_JW_^*^ decreases but *E*_JJ_^*^ increases. Note that in the range −1.4 < μ* < 1.2 the particle–particle interactions dominate . In the case of the AP, we see a deep well in the function  in the range −0.2 < μ* < 0.4 which is correlated with the slight increase in the *E*_JW_^*^. For higher chemical potentials the attractive interactions with the walls become stronger and *E*_JJ_^*^ increases while *E*_JW_^*^ slowly decreases. To summarize, in both pore types, for very low densities and in the dense systems, the interactions with the solid substrates prevail over the particle–particle interactions. Below, we discuss the structural changes observed near the highlighted points above.

**Figure 2:**
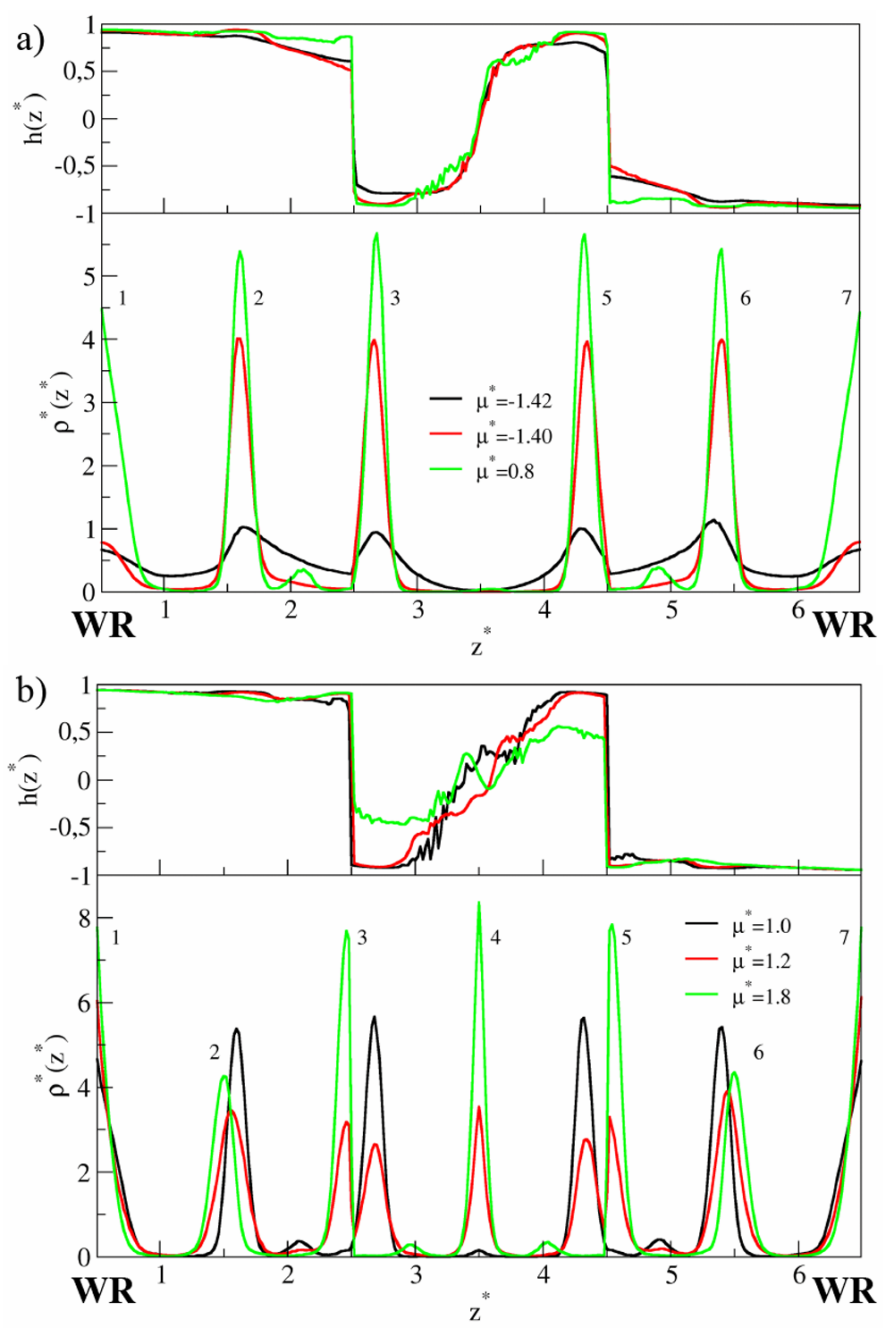
Results for the narrow (H* = 7), symmetrical pore (SP) with weak particle–wall interactions (WC), ε_JW_^*^ = 2. The density profiles (bottom panels) and orientational profiles (top panels) for low (a) and high (b) values of the chemical potential, μ*.

First, we want to discuss the structural transformations in the symmetrical pore resulting from the increase in density. At μ* ≈ −1.4 we found a transition between disordered surface films and the bilayers located at a certain distance from each wall (BL1). We suppose that it can be a first-order transition. However, rigorous proof of this hypothesis is beyond the scope of this study. In Figure 2, the density profiles and orientational profiles *h*(z*) are plotted for selected chemical potentials. The density profiles are symmetrical with respect to the axis located at the pore center. To facilitate the discussion, the peaks corresponding to successive layers are numbered. Initially, the density in the surface region (i.e., in the range of wall potential that is 2σ) is low, and here we found chaotically distributed small clusters of different shapes (layers 1 and 7 in Figure 2a). At μ* ≈ −1.4 we see two high and narrow peaks near each wall (layers 2–3 and 5–6). Notice that the outer peaks of the bilayers (3 and 5) are located outside the force field of the walls. We will show below that these peaks correspond to highly ordered monolayers. Adsorption at the surface is still very low, and the middle part of the pore (unmarked layer 4) is empty.

The morphology of the system results from the competition between adsorption and aggregation. The attractive particle–particle interactions start to dominate which promotes the aggregation (see Figure 1b). However, the impact of the wall enforces the shape of the “giant clusters”, and two bilayers (BL1) are formed parallel to the walls. This is a consequence of the interplay between (i) the system’s geometry, (ii) the relations between ranges of potentials, (iii) possible numbers of neighbors, and (iv) interaction strengths.

In the surface region (layers 1–2 and 6–7), the R-sides of Janus particles are directed mainly toward the walls (see the *h*(*z*) profiles in Figure 2a). However, the particles in the outer layers (3 and 5) of the BL1-structures have opposite orientations. The distance between layers 3 and 5 is close to the range of interactions between particles . Due to the high orientational ordering, these interactions are repulsive. This additionally stabilizes the BL1 structures. In this way, a kind of narrow pore with mutually repelling walls is formed. Adsorption of Janus particles in the middle of such a pore is energetically unfavorable. Therefore, as the chemical potential increases, adsorption at the walls rapidly rises while the pore center remains unfilled (see the density profiles for μ* = 0.8 and 1.0). Simultaneously, the peaks 2–3 and 5–6 become higher. The orientation parameter *h*(z*) tends to 1 in the surface region of the bottom wall, while it is approaching −1 in the third layer (inversely near the top wall).

At the sufficiently dense system, however, a situation changes dramatically (see Figure 2b). For μ* = 1.2, the outer peak of BL1 splits into two lower peaks. One of these double peaks moves toward the wall. The bilayers gradually break down. This enables the formation of the layer in the pore center (layer 4). At μ* = 1.8, the particles accumulate at the walls, at the border of the wall field, and in the middle part of the slit. However, the peaks 2 and 6 become lower and wider. The orientational parameter in the layer 3 (5) is *h* ≈ −0.5 (*h* ≈ 0.5). This reflects the fact that the system gradually transforms into a completely different structure. The evolution of the system structure with increasing density is shown in Figure S1 (SI).

**Figure 3:**
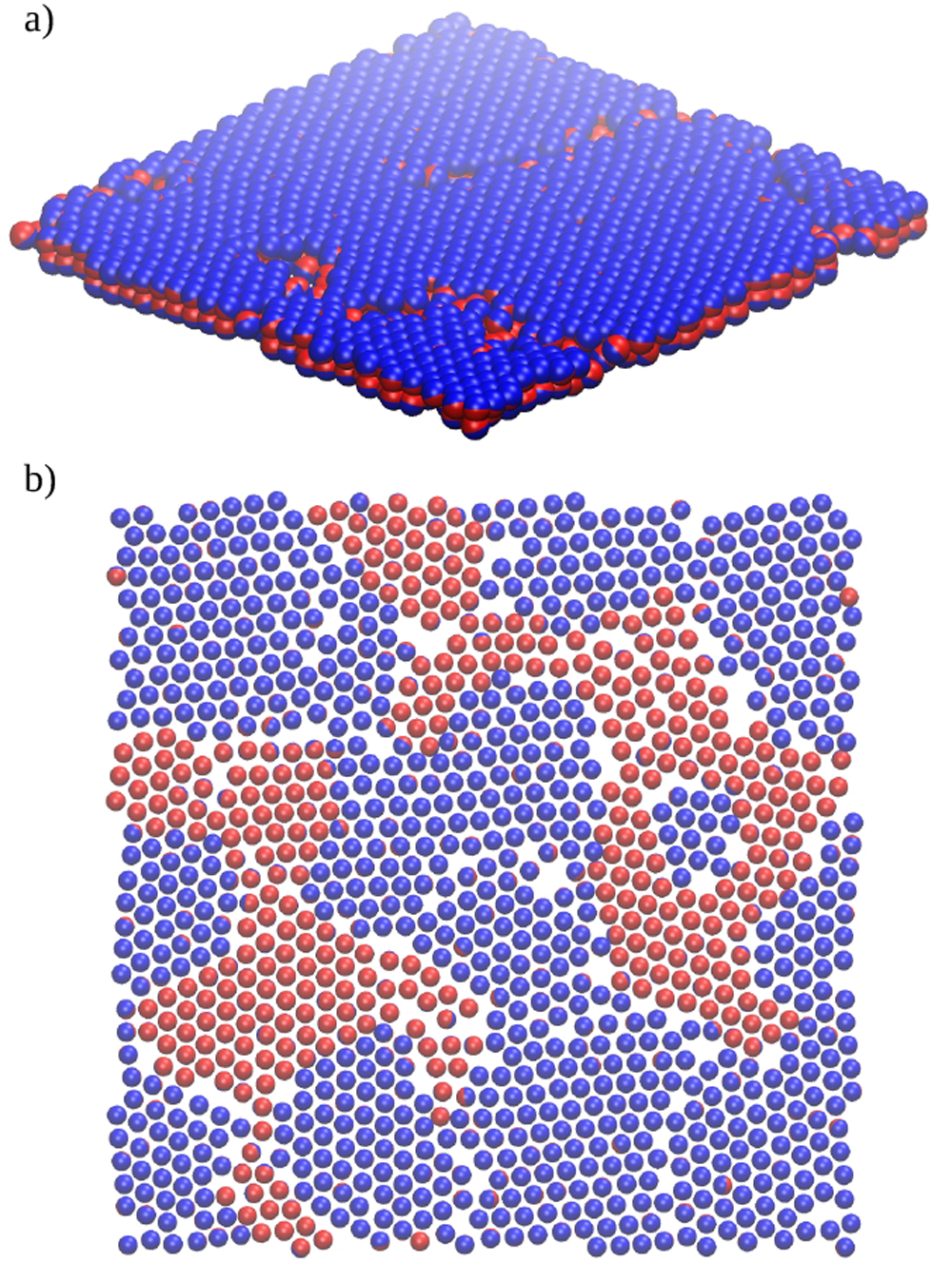
Results for the narrow (H* = 7), symmetrical pore (SP) with weak particle–wall interactions (WC), ε_JW_^*^ = 2. (a) The exemplary configuration of the structure BL1 at μ* = −1.4. (b) A top view of the “split” layer 3 formed at μ* = 1.2.

Let us now discuss the positional and orientational ordering in the selected layers. A representative configuration of the BL1 is shown in Figure 3a for μ* = −1.4. The R- and A-sides are blue and red spheres, respectively. The orientational ordering is evident. We found high positional ordering in the layers forming the BL1 structure. For example, for μ* = −1.4 the fractions of particles forming a hexagonal lattice in the layers 2 and 3 are *P*_*hex*_(2) = 0.78 and *P*_*hex*_(3) = 0.74. Figure 3b presents a top view of the “split” layer 3 formed in a denser pore (μ* = 1.2). In this case, the particles with the same orientations form patches that are slightly *z*-shifted (*h*(z* < 2.5) ≈ 1 and *h*(2.5 < z* < 2.8) ≈ – 1).

Interestingly, for μ* = 1.8 a plastic crystal layer is formed at the middle of the slit. This layer exhibits a significant degree of the positional ordering *P*_*hex*_(4) = 0.56, but there is no orientational ordering at all (*h*(z*) = 0). In this system, the particles begin to form patches of a hexagonal phase also at the walls (*P*_*hex*_(7) = 0.17). Nevertheless, the positional ordering in these layers is much lower compared to that observed in the BL1.

**Figure 4:**
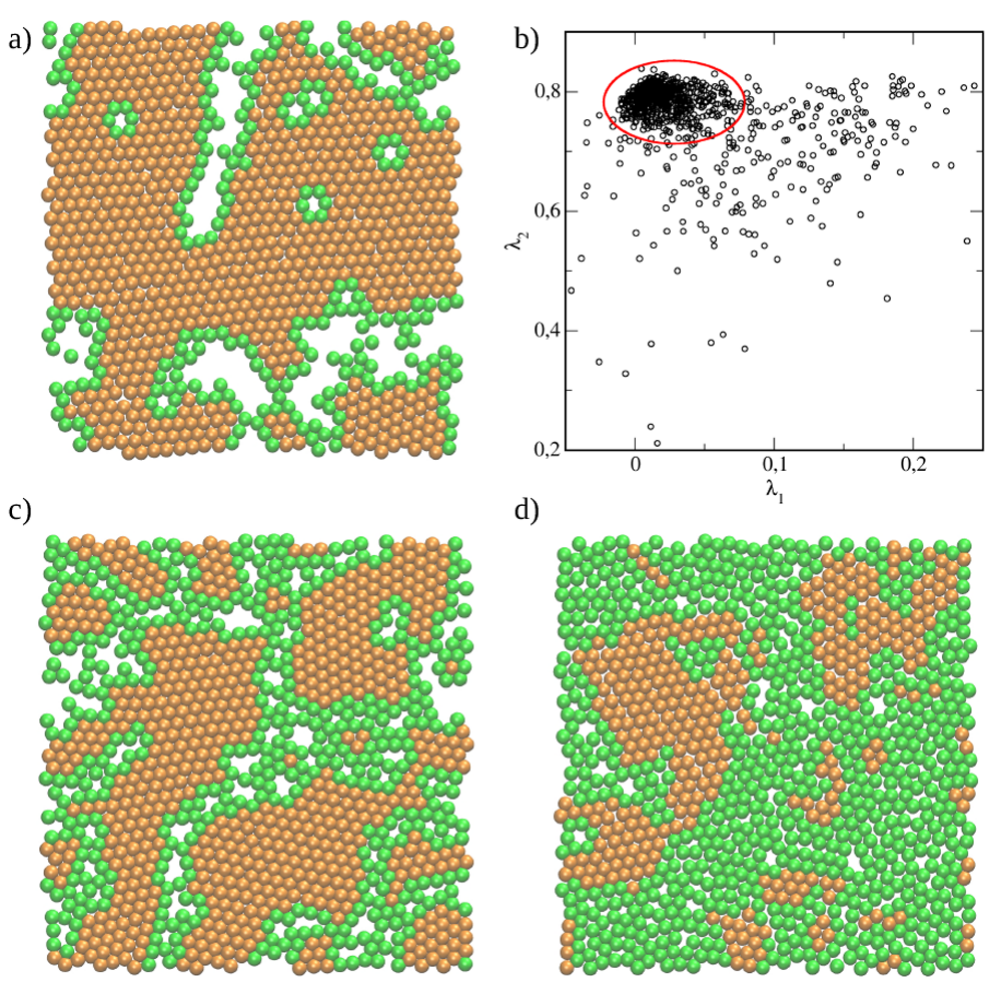
Results for the narrow (H* = 7), symmetrical pore (SP) with weak particle–wall interactions (WC), ε_JW_^*^ = 2. The distribution of different structures within selected layers. The particles which belong to the hexagonal phase are honey spheres while the remaining particles are green spheres. (a) The outer layer of the BL1 (layer 3) at μ* = −1.4. (b) The order parameters in the space (λ_1_, λ_2_). In the bottom panel, the results obtained at μ* = 1.8 are shown: (c) the middle layer (layer 4) and (d) the layer 7 adsorbed at the wall.

To visualize the distribution of different structures within a given layer we show the particles which belong to the hexagonal phase as honey spheres and the remaining particles as green spheres in Figure 4. The picture of the outer layer of the BL1 at μ* = −1.4 is shown in Figure 4a. We see that almost all particles form a hexagonal lattice and the others are located at the interface of crystallites. Figure 4b displays the order parameters in the space (λ_1_, λ_2_). Indeed, the points accumulated in the region corresponding to a hexagonal phase (marked with a red ellipse). The exemplary results obtained at μ* = 1.8 are placed in the bottom panel. The middle layer (layer 4) is presented in part (c), while the distribution of particles adsorbed immediately at the wall is shown in part (d). A low degree of positional ordering at the wall is evident.

**Figure 5:**
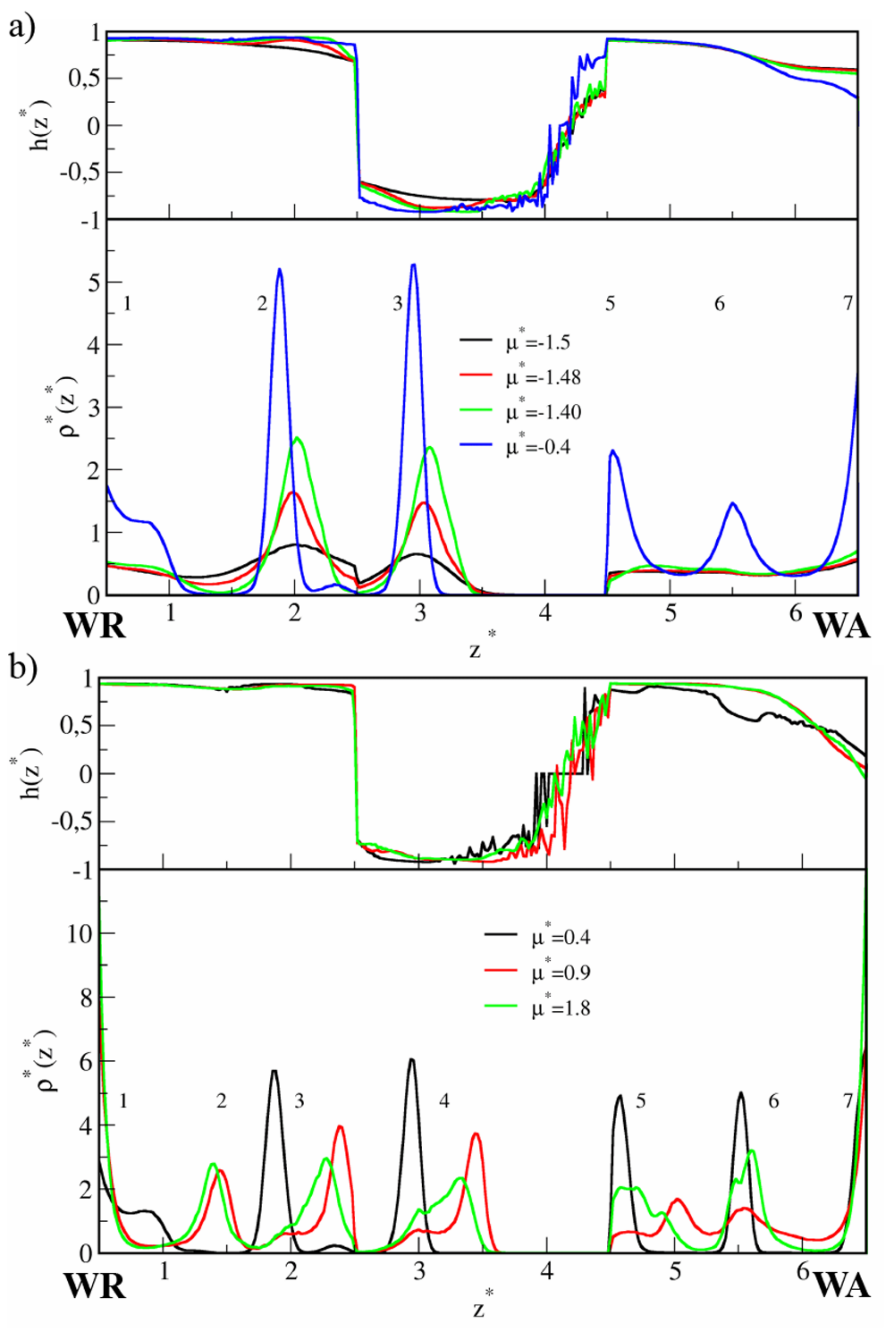
Results for the narrow (H* = 7), Janus-like pore (AP) with weak particle–wall interactions (WC), ε_JW_^*^ = 2. The density profiles (bottom panels) and orientational profiles (top panels) for low (a) and high (b) values of the chemical potential, μ*.

Now let us discuss the structures found in the narrow Janus-like pores. For low densities, adsorption on each wall proceeds almost independently. The surface layer on the wall WR is similar to that observed previously for the symmetrical pore (compare Figure 2a and Figure 5a). In particular, at μ* = −1.4 the highly ordered bilayer (BL1) is formed (layers 2, 3). Adsorption on the other wall (WA) is very low and JPs are chaotically distributed over the surface region. As might be expected, the particles are adsorbed on the WA-surface with the A-sides pointing mainly toward this wall (0.5 < *h*(z*) < 1). A further increase in the density causes the formation of very interesting structures. For −0.2 < μ* < 0.4 three well-pronounced layers are formed (TL1) on the surface WA (see Figure 5b), while the BL1 near the WR remains unchanged. As the distance from the surface WR decreases, the orientation parameter *h*(*z*) gradually changes from 1 to approximately 0.25. Note that this morphology corresponds to the well in the energy *E*_JJ_^*^. At higher densities, the three-layer structure disintegrates and the adsorption directly at the wall WA increases. This transformation is accompanied by a lowering of the orientational order on the WA surface (*h*(*z*) tends to 0). At the same time, the BL1 moves away from the surface WR, the layers become wider, and adsorption on the wall WR also increases (Figure 5b).

**Figure 6:**
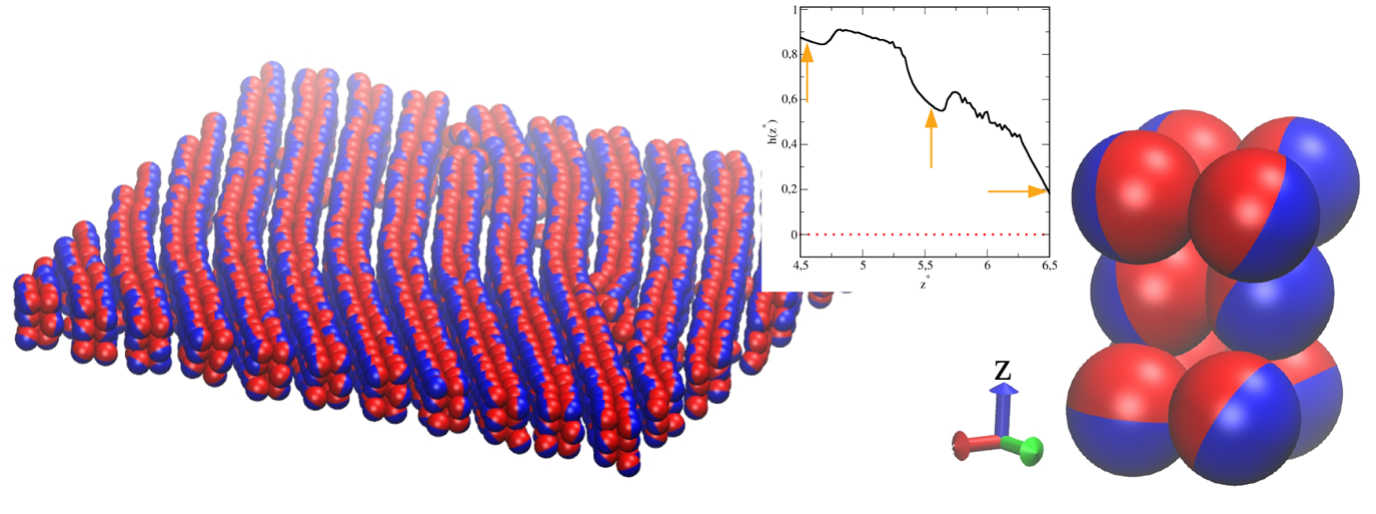
Results for the narrow (H* = 7), Janus-like pore (AP) with weak particle–wall interactions (WC), ε_JW_^*^ = 2. An equilibrium configuration of the TL1-structure. The building block is drawn on the right side. In the inset, the corresponding orientational profile is presented. The arrows indicate the positions of successive maxima in the density profile.

Let us discuss the structure TL1 in more detail. The relevant fragment of the equilibrium configuration with a loose, quasi-lamellar phase is shown in Figure 6. Long, slightly bent stripes are clearly visible in the *xy*-plane. The “low walls” on the surface WA are built of cuboids schematically drawn on the right side. In the inset, we remind how the orientation parameter changes across the TL1- structure. As z* increases, it drops from 0.8 to 0.2. The arrows indicate the positions of successive maxima in the density profile. To show the influence of the particle–wall interaction strength on the structure of the system, we also performed simulations for ε_JW_^*^ = 4 (SC). In the case of strong particle–wall coupling, all estimated isotherms are continuous, and adsorption is greater than that discussed previously. As expected, isotherms obtained for the SC-pores are shifted toward lower chemical potentials compared to those for the WC-pores. The particle–walls interactions always dominate, *E*_JW_^*^ < *E*_JJ_^*^, and the energy changes with μ* are continuous.

**Figure 7:**
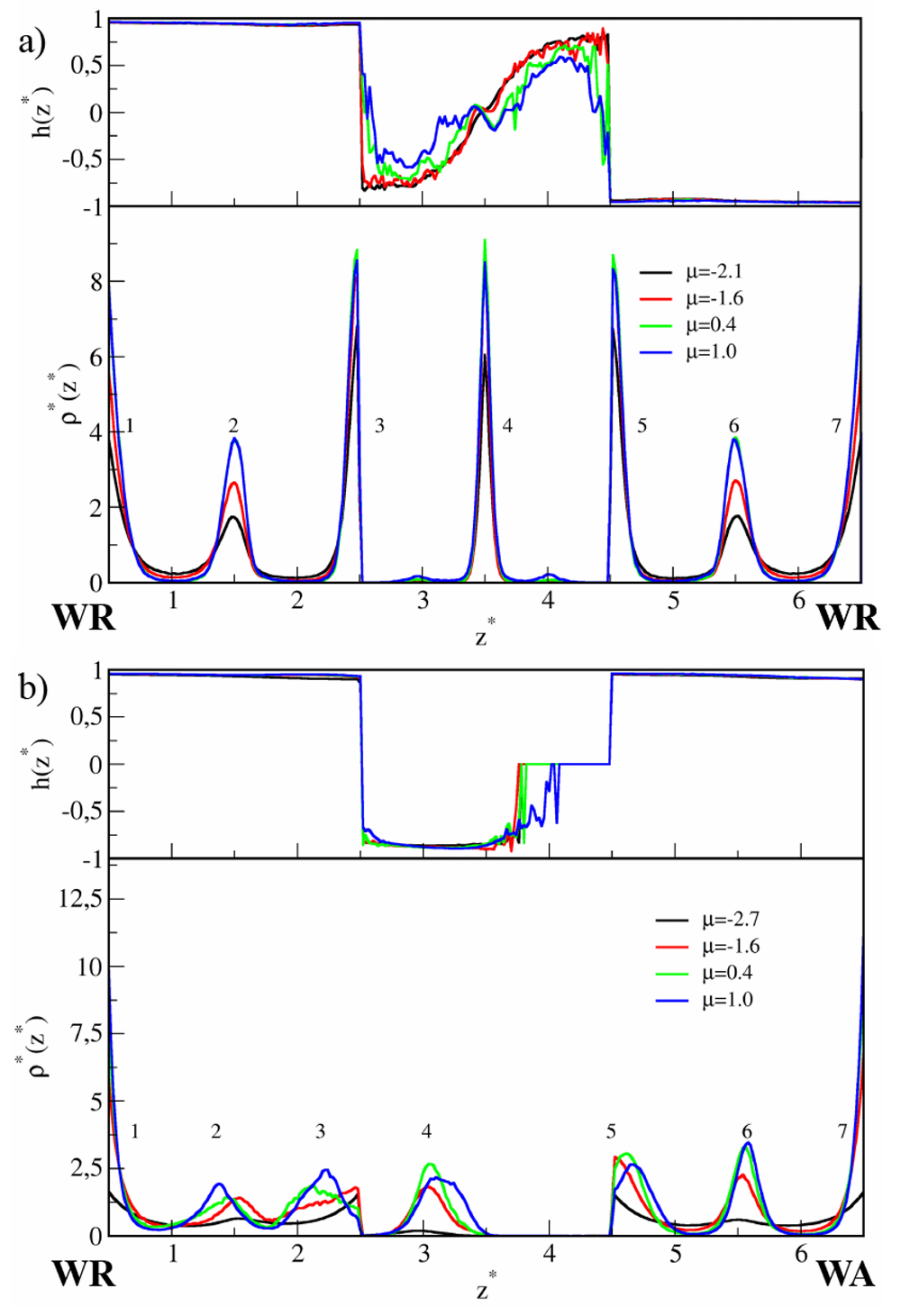
Results for the narrow (H* = 7) pores with strong particle–wall interactions (SC), ε_JW_^*^ = 4. The density profiles (bottom panels) and orientational profiles (top panels) for (a) symmetrical (SP) and (b) Janus-like (AP) pores and selected values of the chemical potential, μ*.

Figure 7 depicts density profiles for the SC-slits and relatively high chemical potentials. We start with the discussion of a situation in the symmetrical pore (Figure 7a). The structure of the system differs from that obtained for the slit with weaker walls (compare with Figure 2). First, we observe large adsorption at the walls, even at low chemical potentials. Second, there are no bilayers (BL1), typical for the systems with ε_JW_^*^ = 2. The particles accumulate closer to the walls; three layers are formed in the range of the potential of each wall. The outer layers (layers 3 and 5) do not interact with each other. As before, at low chemical potentials the middle part of the pores remains empty (not shown here), while at higher μ*, an additional layer forms in the center of the pore (cf. Figure, 2b). It is noticeable that stronger confining forces prevent the previously observed 2D ordering in layers 2 and 6 (see Table 1 in Supporting Information).

The density profiles for the Janus-like pores are plotted in Figure 7b. At the wall WA, an increase in the strength of particle–wall interactions does not have a qualitative impact on the morphology of the surface layer. As previously, the three-layer film TL1 is observed. This is reflected by three peaks in the density profiles. On the other hand, a completely different structure is observed at the WR wall. We see here a series of peaks near the wall: the very high peak at the wall, two much lower ones in the surface region, and the peak outside the field of the wall (z* = 2.5). It should be pointed out that there is no 2D positional ordering within these layers.

To sum up, for weak particle–wall interactions, the unique BL1-structures are formed near the WR-surfaces, while on the WA-walls the special TL1-structures are observed. We found high degrees of the 2D positional ordering in the layers 2–6 in the BL1. If the particle–wall interactions are strong, the bilayers BL1 disappear, and the adsorption in the region closest to the surface increases. Moreover, the 2D ordering in the internal layers decreases but slightly increases at the walls. As expected, for stronger interactions with the surfaces the orientational ordering near the walls increases. This confirms our hypothesis that some unique structures appear only for relatively weak walls. Strong surfaces impede positional ordering in the inner layers (see SI, Table 1). In this case, the structure of the adsorbed fluid is considerably imposed by the walls.

### Adsorption and Self-Assembly in the Wide Slits with H* = 11

**Figure 8:**
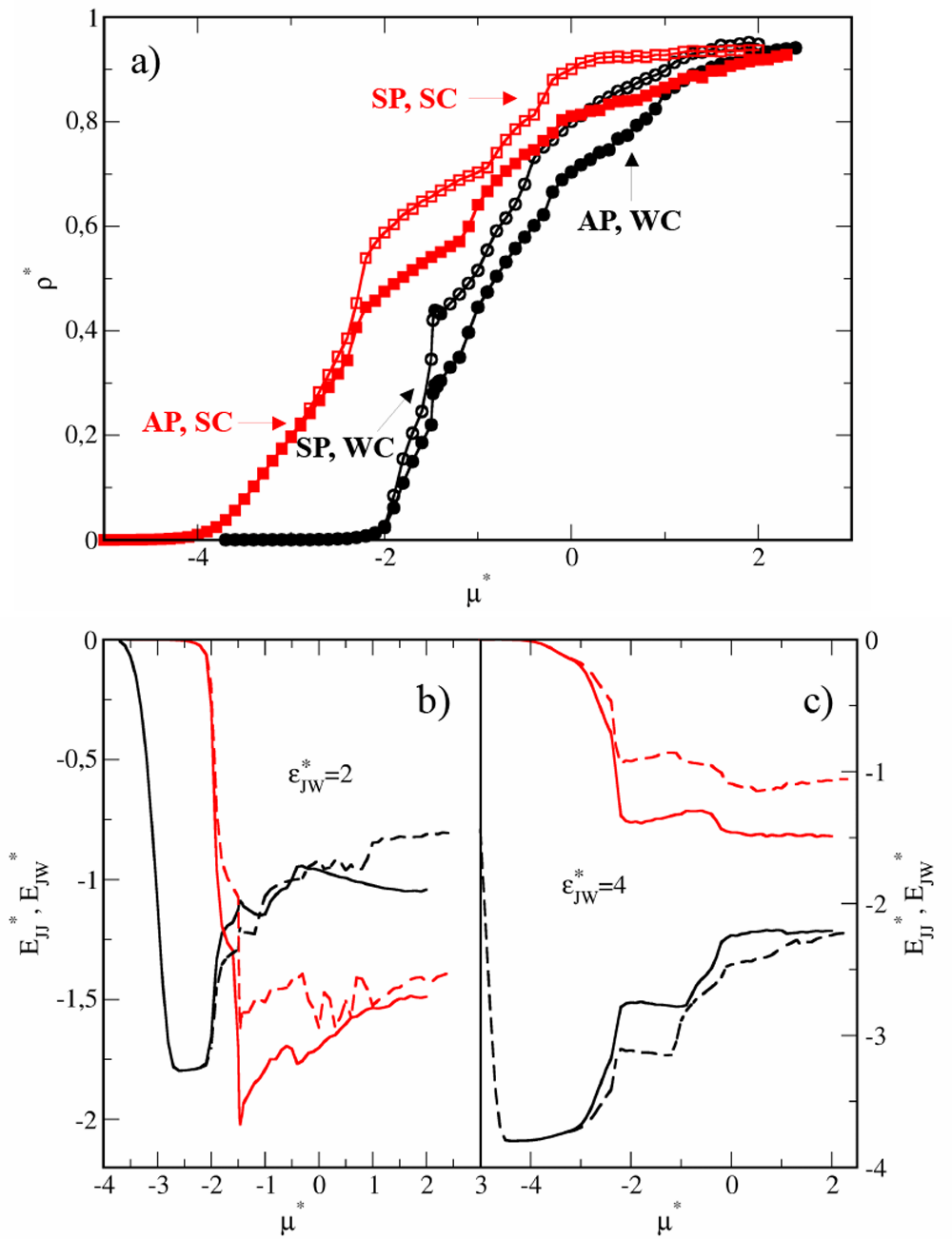
Results for the wide pores (H* = 11). (a) Adsorption isotherms for the case WC, ε_JW_^*^ = 2 (black circles) and SC, ε_JW_^*^ = 4 (red squares) in symmetrical pores SP (open symbols) and Janus-like pores AP (filled symbols). The energies *E*_JJ_^*^ (red lines) and *E*_JW_^*^ (black lines) in symmetrical pores (solid lines) and Janus-like pores (dashed lines) for the case WC, ε_JW_^*^ = 2 (b) and SC, ε_JW_^*^ = 4 (c).

In this section, we discuss results obtained for wider slits. The isotherms and the energies of interactions for these systems are shown in Figure 8. We focus here on the pores with weakly interacting walls (WC, ε_JW_^*^ = 2). Similar to narrower slits, there is a jump in adsorption at μ* ≈ – 1.4, but then adsorption rises continuously. In the case of symmetrical pores, the energy landscape is only slightly different compared to that for the narrow slits. However, for the Janus-like slits the differences are more significant, namely, in the range −0.2 < μ* < 1, a series of alternating extrema is visible in the plot . This suggests the existence of a new structure TL2, which we will discuss below. Example configurations of systems with different densities are shown in Figure S2 (SI).

**Figure 9:**
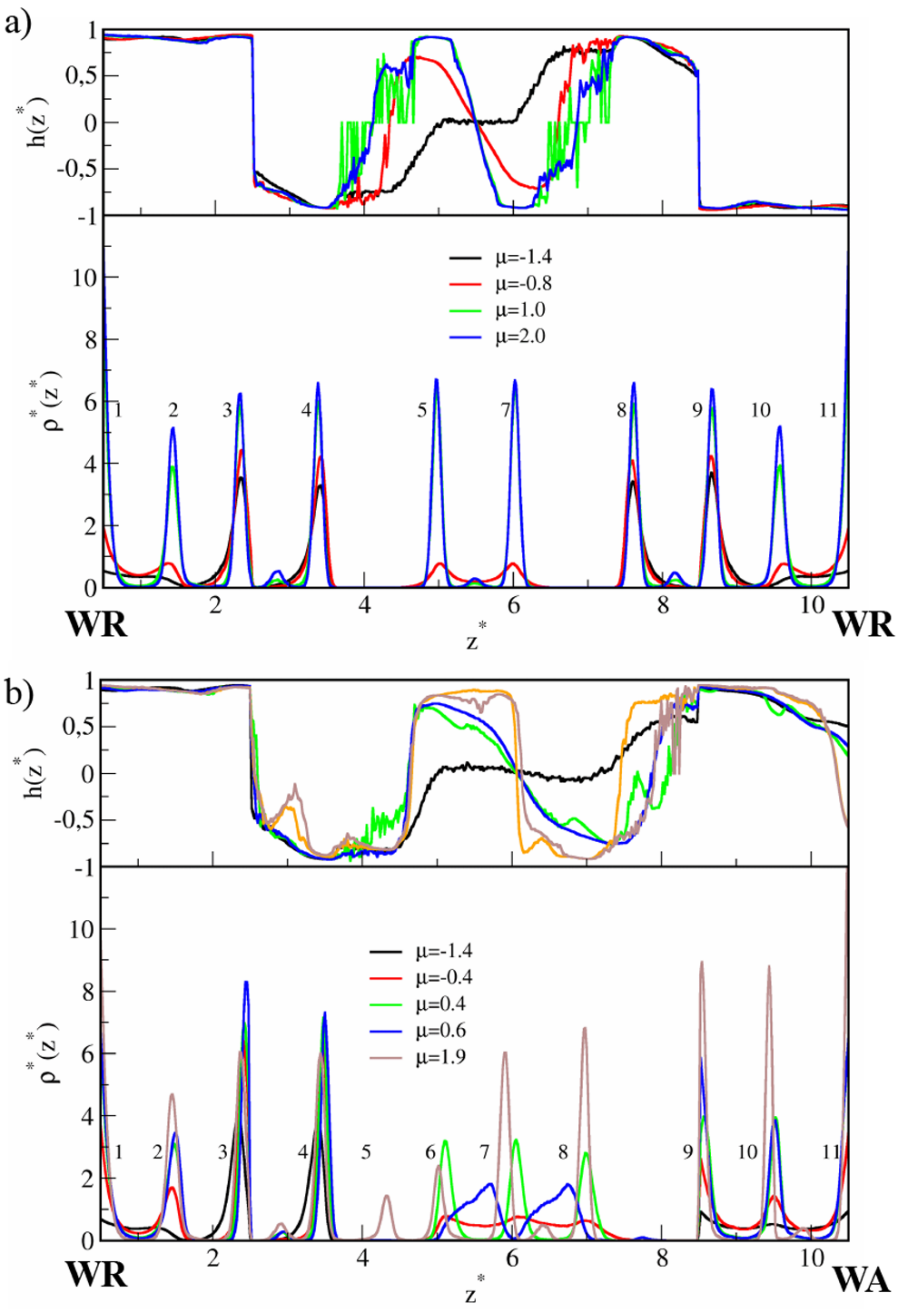
Results for the wide (H* = 11) pores with weak particle–wall interactions (WC), ε_JW_^*^ = 2. The density profiles (bottom panel) and orientational profiles (top panel) for (a) symmetrical (SP) and (b) Janus-like (AP) pores, and selected values of the chemical potential, μ*.

In the case of symmetrical pores, the morphology of the wide slit is similar to that in the narrow one (see Figure 9). In particular, the ordered BL1 structures are observed at each surface. However, for higher densities, two layers are formed in the center of pores (BL2) instead of one, and four layers near the walls. The layers in the structures BL1 and BL2 exhibit a considerable degree of the 2D ordering (see Table 2 in Supporting Information).

For the Janus-like slit, a picture of structure transformations is more interesting. The morphology of the layer near the wall WR changes in the same way as in the wide symmetrical pore (compare parts a and b). Moreover, the layers formed at the WA walls in the narrow and wide pores are quite similar. In both cases, the quasi-lamellar structures TL1 are observed. However, in the center of the slit, a very special structure (TL2) is formed. At μ* = 0.4 three well-pronounced identical peaks are visible, indicating the existence of “the three-layer cluster” in the region 4.8 < z* < 5.2. As the chemical potential increases to μ* = 0.6, these peaks spread into two wide peaks in the density profile indicating that the structure TL2 is falling apart. Thus, the structure TL2 transforms gradually into the bilayer. Surprisingly, at μ* = 1.3 (not shown in Figure 9b) three maxima appear again and remain visible with a further increase in density also at μ* = 1.9. It is interesting that at certain intermediate densities the structure TL2 cannot be formed but it is stable at high densities.

**Figure 10:**
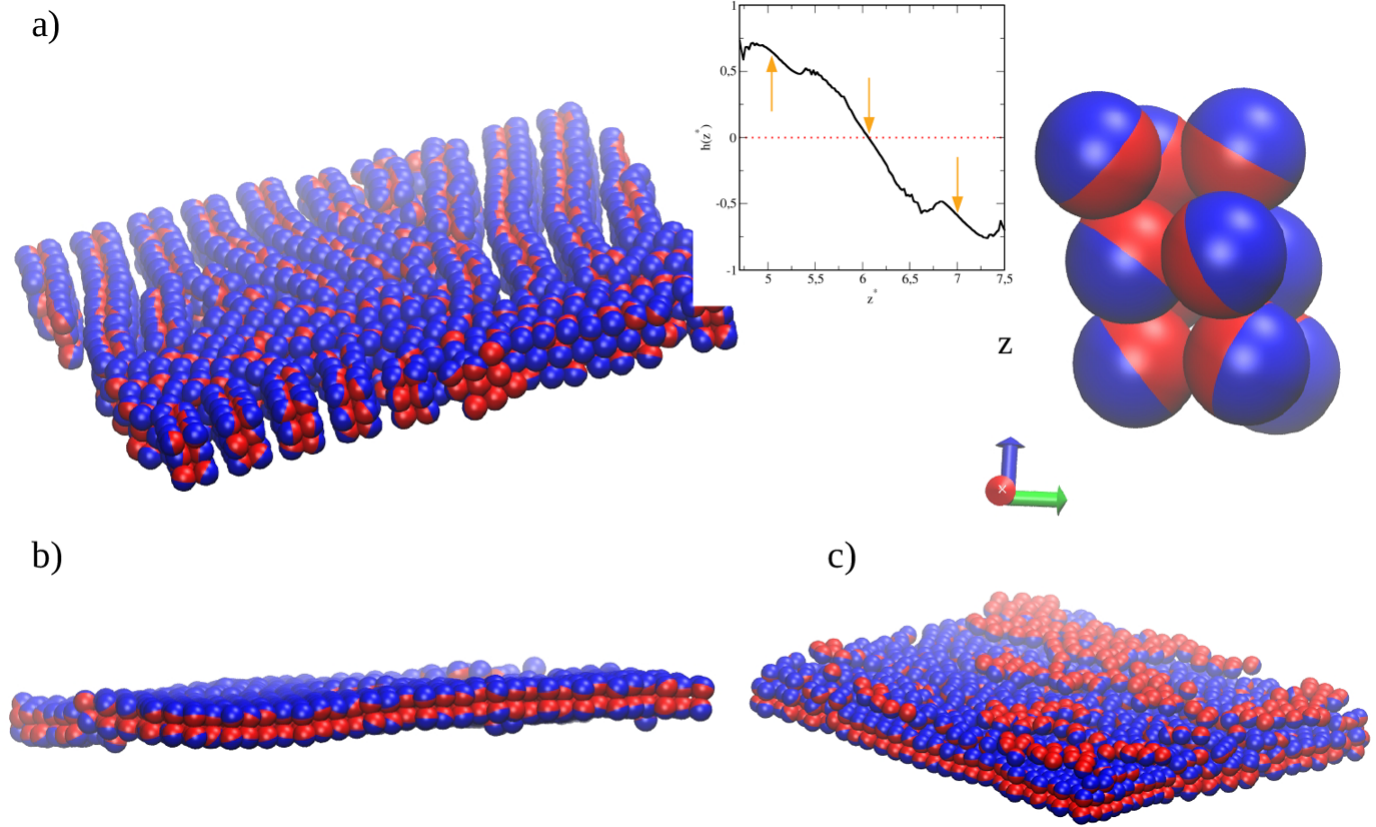
Results for the wide (H* = 11), Janus-like (AP) pore with weak particle–wall interactions (WC), ε_JW_^*^ = 2. (a) An equilibrium configuration of the TL2-structure, the building block, and the corresponding orientational profile (inset) for μ* = 0.4. The arrows indicate the positions of successive maxima in the density profile. (b) The bilayer observed for μ* = 0.6 and (c) the structure in the region 4 < z* < 8 at μ* = 1.9.

We analyzed the structure TL2 formed inside the wide Janus-like pore and found that it is considerably different from the phase of the TL1-type built at the wall WA. As we show in Figure 10a, the structure TL2 is built of the cuboids in which orientations of the Janus particles are significantly different than those in the structure TL1 (compare insets in Figure 6 and Figure 10a). For the TL2 structure, the orientation parameter *h*(z*) decreases from 0.75 to −0.75 in the *z*-direction. The particles in the outer layers of the TL2 are almost oppositely oriented. At the bottom, we present the fuzzy and pleated bilayer observed for μ* = 0.6 and the highly rough structure that appears in the region 4 < z* < 8 at μ* = 1.9.

**Figure 11:**
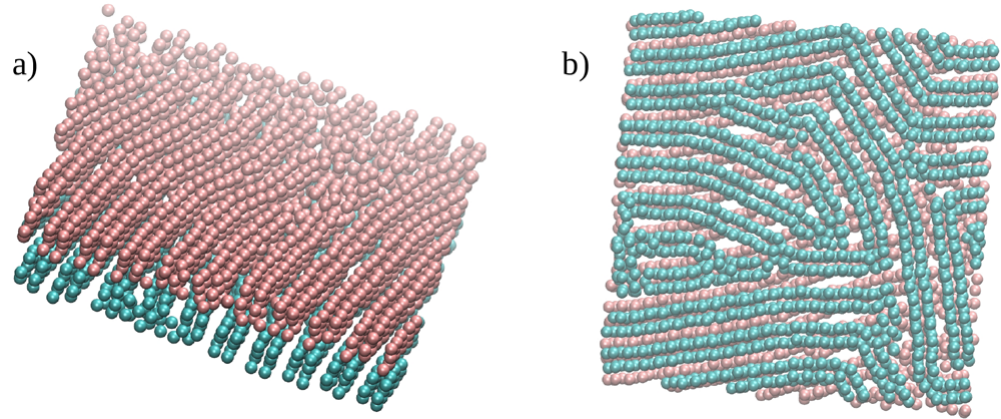
Results for the wide (H* = 11), Janus-like (AP) pore with weak particle–wall interactions (WC), ε_JW_^*^ = 2. The arrangement of structures TL1 (pink spheres) and TL2 (blue spheres) relative to each other: (a) a side view, (b) a view from above.

In Figure 11 we present the arrangement of structures TL1 (pink spheres) and TL2 (blue spheres) relative to each other. Note that the orientations of the “strips” are not correlated. To complete our discussion we analyzed the results for ε_JW_^*^ = 4 (Figures S3 and S4). For symmetrical pores, structures similar to those described above are formed. However, the successive layers exhibit a considerably lower degree of ordering (Table 2 in Supporting Information). For example, in the case of ε_JW_^*^ = 2, *P*_*hex*_(3) = 0.83 and *P*_*hex*_(4) = 0.78 (μ* = −0.8), while for ε_JW_^*^ = 4, *P*_*hex*_(3) = 0.58 and *P*_*hex*_(4) = 0.55 (μ* = −0.9). It is interesting that at a high density (μ* = 1.9) we found a certain degree of 2D ordering near the walls (*P*_*hex*_(1) = 0.21 and *P*_*hex*_(2) = 0.20). Also in Janus pores, the surface layers are very similar to those observed for weaker walls (cf. Figure 9b and Figure S4 in SI). Thus, the effect of increasing the wall strength on the structure of the system is similar to that observed in narrow pores (see Table 2 in Supporting Information).

## 4 Conclusions

We have studied the self-assembly of amphiphilic Janus particles confined between two solid surfaces using Monte Carlo simulations in the grand canonical ensemble. We have considered Janus particles consisting of attractive and repulsive hemispheres confined in two types of slits: (i) symmetrical pores (SP), with identical “walls” attracting the repulsive parts of JPs, and (ii) antisymmetrical, Janus-like slits (AP), in which one “wall” attracts the repulsive hemispheres of JPs, while the other attracts their attractive parts. We have focused on the impact of the density of Janus particles on the system morphology. So far, this issue has not been systematically studied.

We have estimated the adsorption isotherms for narrow (H* = 7) and wide (H* = 11) pores with weak WC,  and strong SC,  walls. In all cases, we analyzed the structural changes resulting from the increase in the density of particles adsorbed in the pores. To characterize the system morphology, we have calculated the density profiles of JPs along the *z*-axis, ρ*(z*), the orientation profiles, *h*(z*), and the parameters measuring the degree of positional ordering in the system (λ_1_, λ_1_, *P*_*i*_).

In general, the different self-assembly scenarios are due to the geometry of the system and the contributions of particle–particle and wall–particle interactions to the total energy. This results in the formation of structures that cannot be formed in the bulk phases.

The most interesting results are obtained for relatively weak interactions with the walls (WC). In this case, we observe a complex interplay between aggregation and adsorption in the surface regions. A special geometry of the slits enforces the formation of layers parallel to the walls, instead of micelle-like clusters. In the symmetrical pores, at moderate densities, the characteristic bilayers are formed near each wall. These BL1-structures are located at a certain distance from the walls. The outer layers of the BL1s lie beyond the force field of the surface. The layers exhibit high orientational ordering. In the surface region, the R-sides of Janus particles are directed mainly toward the closest wall, while the particles in the outer layers have opposite orientations. In this way the adsorbed particles create a kind of pore with mutually repulsive walls in the slit’s center. Further adsorption of Janus particles inside this pore becomes energetically unprofitable. Indeed, the density at the middle part of the slit is extremely low. However, at a sufficiently high density, the system morphology changes, the bilayers disappear, particles accumulate at the walls, and an additional layer is formed in the pore center. We have analyzed the 2D ordering in these structures and detected the existence of hexagonal lattices, in some cases. In particular, the BL1 has a high degree of the hexagonal ordering, but the layers at the walls are completely disordered.

In the Janus-like pore, we found two very interesting, three-layer structures, namely, the structure TL1 at the WA-walls and the structure TL2 in the middle part of the wide slit (H* = 11). The structures resemble loose, quasi-lamellar phases in which the “stripes” are built of cuboids. The orientations of particles in the cuboids belonging to the TL1 layers are different from the orientations in the building blocks of the TL2 layers. We carried out also simulations for stronger particle–wall interactions (SW, ε_JW_^*^ = 4). In this case, the bilayers BL1 disappear, and the adsorption at the walls increases. Moreover, the 2D ordering in the internal layers decreases but slightly increases at the walls. As expected, for stronger interactions with the surfaces the orientational ordering near the walls increases. Our simulations show that the morphology of the system is highly dependent on the strength of the force field generated by the walls. Our simulations demonstrated how the self-assembly of amphiphilic Janus nanoparticles can be controlled by the nature of the pores and the particle’s density. These findings may be expected to contribute to the development of novel applications involving anisotropic building blocks. For example, the design of the chemical nature of the walls can be one of the key factors controlling the assembly. Furthermore, the essential role of spatial confinement on the positional and orientational ordering revealed here may be useful for understanding the behavior of naturally occurring anisotropic colloids, such as proteins, confined by interfaces.

We hope that our study has provided new knowledge about self-assembly in closed systems that can be applied to the design of unique structures composed of Janus particles.

## Supporting Information Available

Figure S1: Additional snapshots for narrow symmetrical pores. Figure S2: Additional snapshots for wide Janus-like pores. Figures S3, S4: Density and orientational profiles for wide pores with strong particle–wall interactions. Tables S1, S2: Tabulated data for fractions of particles forming a hexagonal lattice in different layers. (PDF)
