# Supplementary material for: Amphiphilic Janus Particles Confined in Symmetrical and Janus-Like Slits
Source: ACS Omega. 2023 May 16;8(21):18863–73. doi: 10.1021/acsomega.3c01180 (PMC10233691; doi:10.1021/acsomega.3c01180)
Supplement: Supplementary file 1 — ao3c01180_si_001.pdf [file ao3c01180_si_001.pdf]

Amphiphilic Janus particles confined in symmetrical and Janus-like slits
Supporting Information

Łukasz Baran,[*,†] Małgorzata Borówko,[†] Wojciech Rżysko,[†] and Jakub Smołka[‡]

†*Department of Theoretical Chemistry, Institute of Chemical Sciences, Faculty of Chemistry, Maria-Curie-Sklodowska University in Lublin, Pl. M Curie-Sklodowskiej 3, 20-031 Lublin, Poland*

‡*Department of Computer Science, Lublin University of Technology, Nadbystrzycka 36B, 20-618 Lublin, Poland*

E-mail: lukasz.baran@mail.umcs.pl

a)

b)

c)

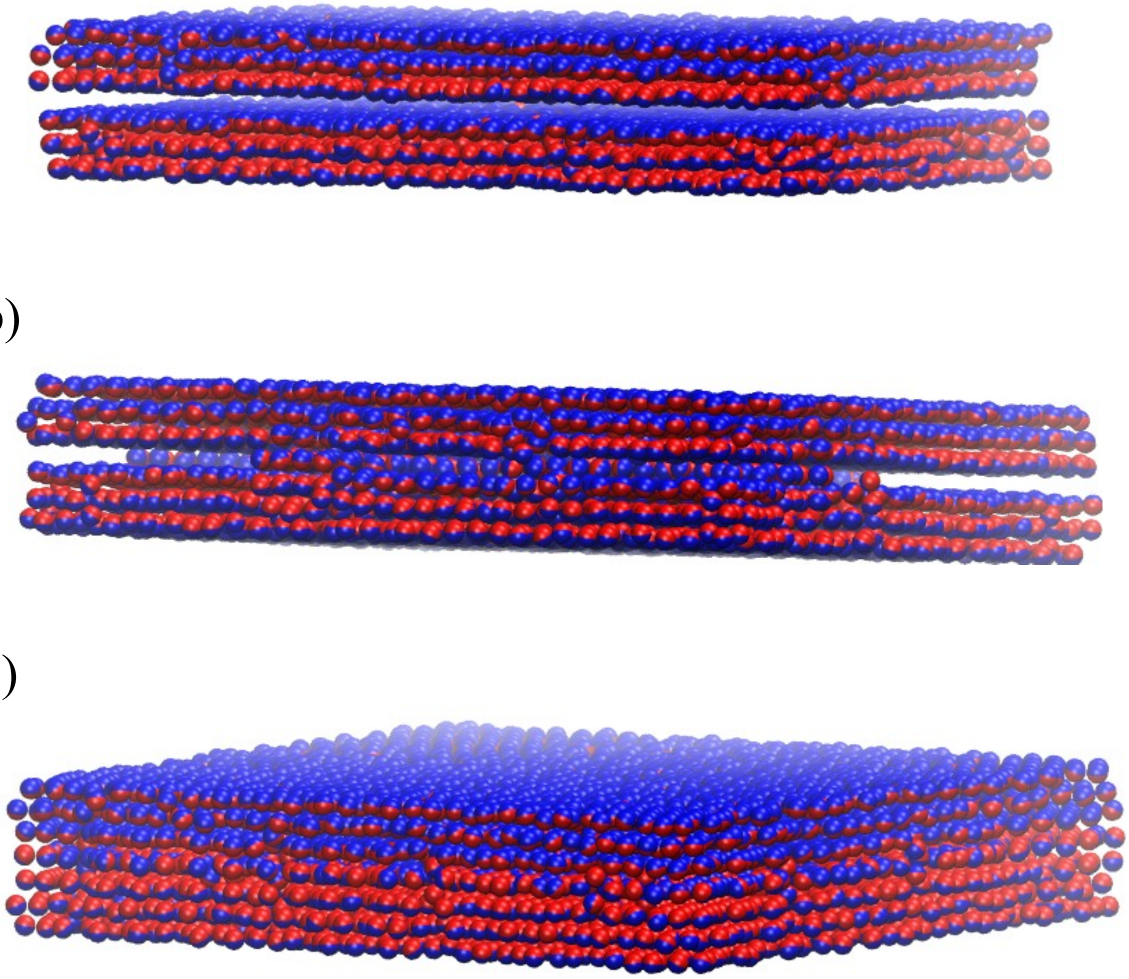

Figure S1: Results for the narrow ($H^* = 7$), symmetrical pore (SP) with weak particle-wall interactions (WC), $\varepsilon^*_{JW} = 2$. Example equilibrium configurations for the following values of the chemical potential, μ^*: (a) 1.0, (b) 1.2, and (c) 1.8 (cf. Fig, 2b).

a)

b)

c)

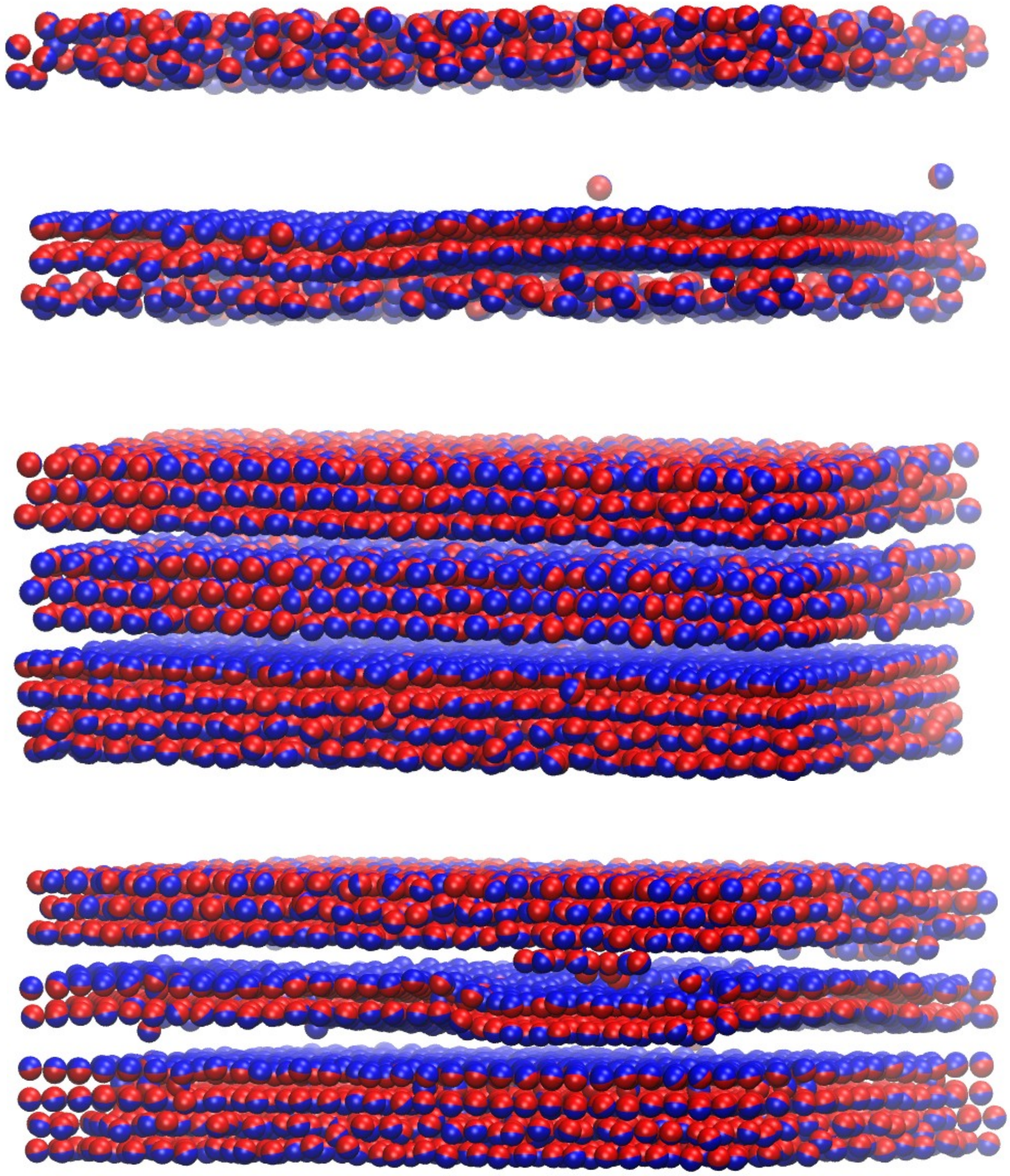

Figure S2: Results for the wide ($H^* = 11$) Janus- like pore (AP) with weak particle-wall interactions (WC), $\varepsilon^*_{JW} = 2$. Example equilibrium configurations for the following values of the chemical potential, μ^*: (a) -1.4, (b) 0.4, and (c) 0.6 (cf. Fig, 9b).

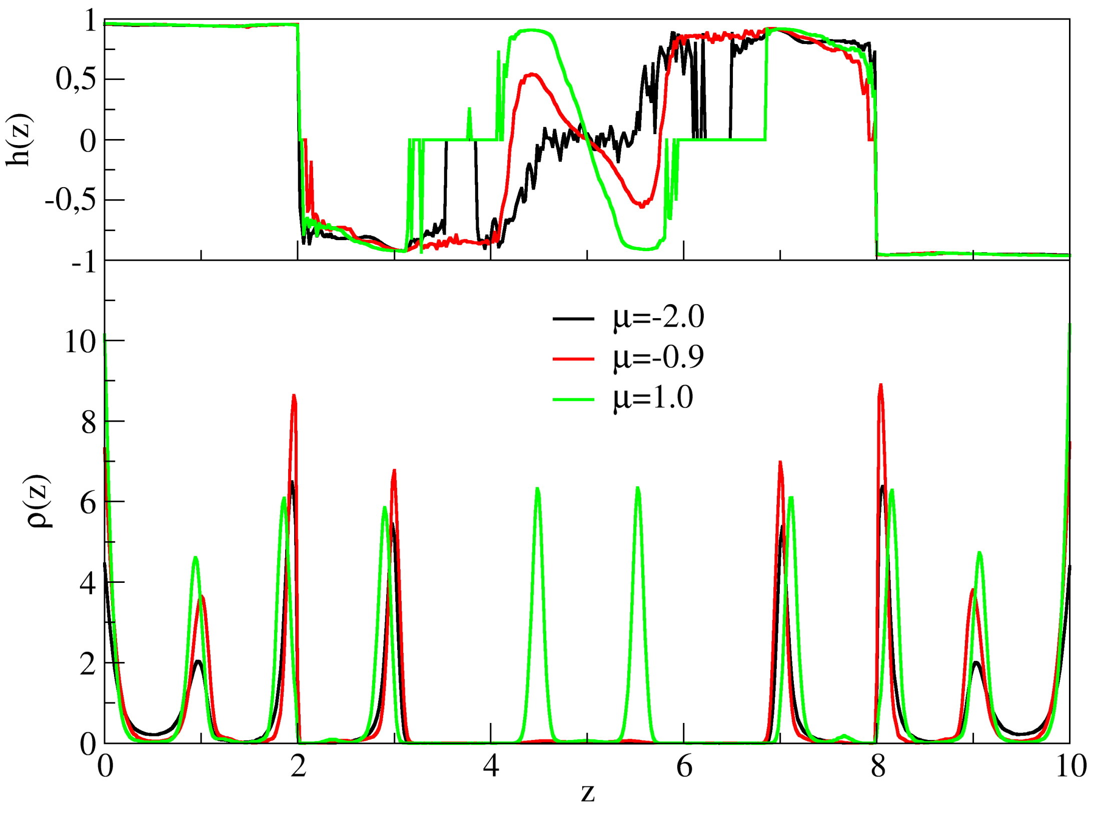

Figure S3: The density profiles (bottom panel) and orientational profiles (top panel) for symmetrical pores and selected values of the chemical potential, μ^*. Remaining parameters: $H^* = 11$ and $\varepsilon^*_{JW} = 4$.

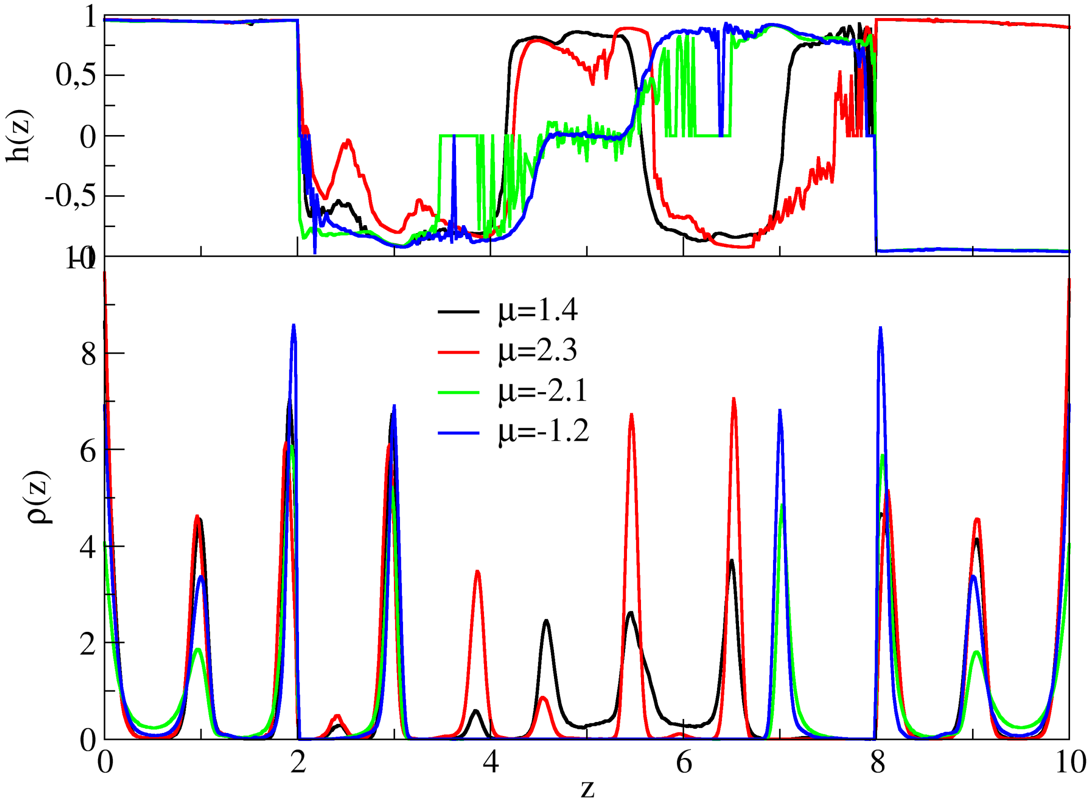

Figure S4: The density profiles (bottom panel) and orientational profiles (top panel) for Janus-like pores and selected values of the chemical potential, μ^*. Remaining parameters: $H^* = 11$ and $\varepsilon^*_{JW} = 4$.

Table S1: Fractions of particles forming a hexagonal lattice in successive layers, P_{hex} in the narrow pores ($H^* = 7$).

ε^*_{JW}	SP/AP	μ^*	1	2	3	4	5	6	7
		-1.4	0.	0.78	0.74	-	0.67	0.72	0.
2	SP	0.9	0.	0.63	0.62	-	0.54	0.59	0.01
		1.8	0.39	0.44	0.62	0.65	0.66	0.39	0.32
		-1.4	0.	0.67	0.64	-	-	-	-
2	AP	0.4	0.	0.64	0.68	0.	0.	0.	0.
		0.9	0.	0.60	0.57	0.55	0.	0.	0.
		1.8	0.	0.	0.27	0.29	0.07	0.18	0.10
		-2.1	0.	0.	0.14	0.10	0.16	0.	0.
4	SP	-1.6	0.	0.	0.43	0.40	0.43	0.	0.
		0.4	0.01	0.04	0.51	0.60	0.52	0.08	0.02
		1.0	0.05	0.08	0.54	0.55	0.51	0.08	0.05
4	AP	1.0	0.	0.	0.28	0.25	0.10	0.16	0.12

Table S2: Fractions of particles forming a hexagonal lattice in successive layers, P_{hex} in the wide pores ($H^* = 11$).

ε^*_{JW}	SP/AP	μ^*	1	2	3	4	5	6	7	8	9	10	11
		-2.1	-	-	0.71	0.56	-	-	-	0.72	0.80	0.	0.
2	SP	-1.6	-	-	0.83	0.78	-	-	-	0.73	0.76	0.	0.
		1.0	0.	0.	0.57	0.50	0.67	-	0.66	0.50	0.51	0.	0.
		2.0	0.25	0.23	0.41	0.42	0.59	-	0.59	0.55	0.58	0.19	0.20
		-1.5	-	-	0.02	0.02	-	-	-	-	-	-	-
2	AP	-1.4	-	-	0.76	0.735	-	-	-	-	-	-	-
		0.4	0.	0.	0.61	0.59	-	-	-	-	-	-	-
		0.6	-	-	-	-	-	0.74	0.70	-	-	-	-
		1.0	0.02	0.02	0.60	0.58	-	-	0.70	0.67	0.11	0.72	0.59
		1.9	0.21	0.20	0.51	0.48	-	-	0.55	0.55	0.34	0.57	0.52
		-2.6	0.	0.	0.77	0.45	-	-	-	-	-	-.	-
4	SP	-2.0	0.	0.	0.73	0.46	-	-	-	0.47	0.65	0.	0.
		-0.9	0.	0.02	0.58	0.55	-	-	-	0.60	0.65	0.01	0.02
		0.0	0.05	0.07	0.57	0.50	0.67	-	0.65	0.51	0.55	0.07	0.08
		2.0	0.22	0.31	0.68	0.65	0.71	-	0.68	0.67	0.69	0.36	0.32
		-2.1	0.	0.	0.72	0.40	-	-	-	-	-	-	-
4	AP	-1.2	0.	0.	0.68	0.65	-	-	-	-	-	-	-
		2.3	0.20	0.22	0.49	0.47	0.	0.	0.89	0.88	0.57	0.73	0.50
